# Cooling Concepts: Alternatives to Air Conditioning for a Warm World

**DOI:** 10.1289/ehp.121-a18

**Published:** 2013-01-01

**Authors:** Richard Dahl

**Affiliations:** **Richard Dahl**, a freelance writer in Boston, has contributed to *EHP* since 1995. He also writes periodically for the Massachusetts Institute of Technology.

Two massive power blackouts occurring on consecutive days last summer in India^^1^^ have highlighted the difficulties developing nations face when vulnerable power grids are taxed by the growing use of air conditioners.

One factor in the blackouts is a weak monsoon season in India, which resulted in below-normal water levels at some hydroelectric dams and less electricity to go around.^^1^^ Escalating consumer demand for air conditioners likewise may be implicated. Catherine Wolfram, co-director of the Energy Institute at Haas School of Business, University of California, Berkeley, points to data showing that the increase in energy demand between the hottest and coldest months of the year among the 16 million residents of Delhi, India, more than doubled between 2000 and 2009.^^2^^ In a 2012 essay on this research she concluded, “A large part of the explanation for this is that air conditioner sales have increased dramatically.”^^3^^

**Figure:**
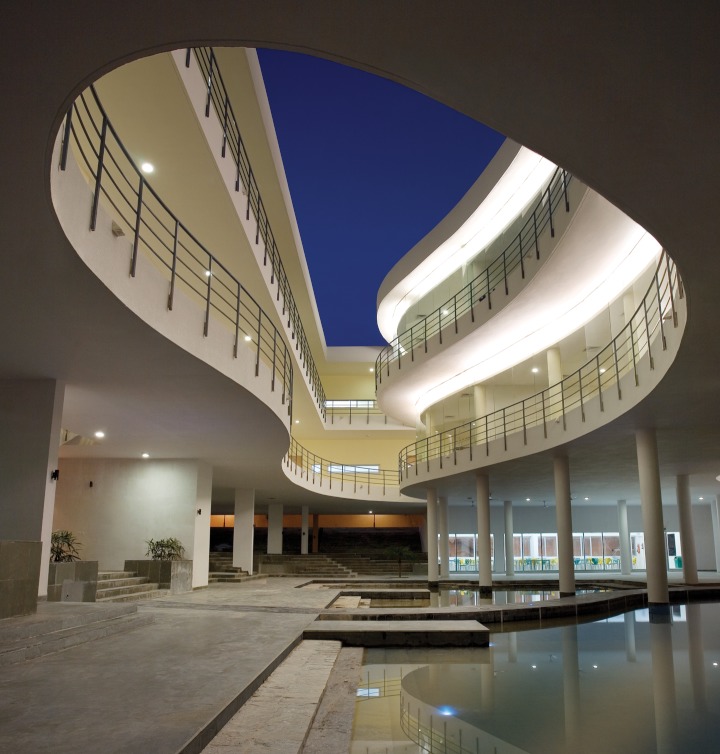
Pearl Academy in Jaipur, India, features a sunken pool, inspired by traditional Rajasthani architecture, that takes advantage of the thermalregulating properties of the ground. © André J Fanthome

The developing world is home to most of the world’s hottest and fastest-growing cities—38 of the world’s 50 largest cities are in developing countries, and most of the 30 warmest of these cities are in developing countries.^^4^^ These countries also have a rapidly expanding middle class that can now afford the amenities that citizens in the developed world have long taken for granted.^^5^^ High on that list is the air conditioner.

**Figure:**
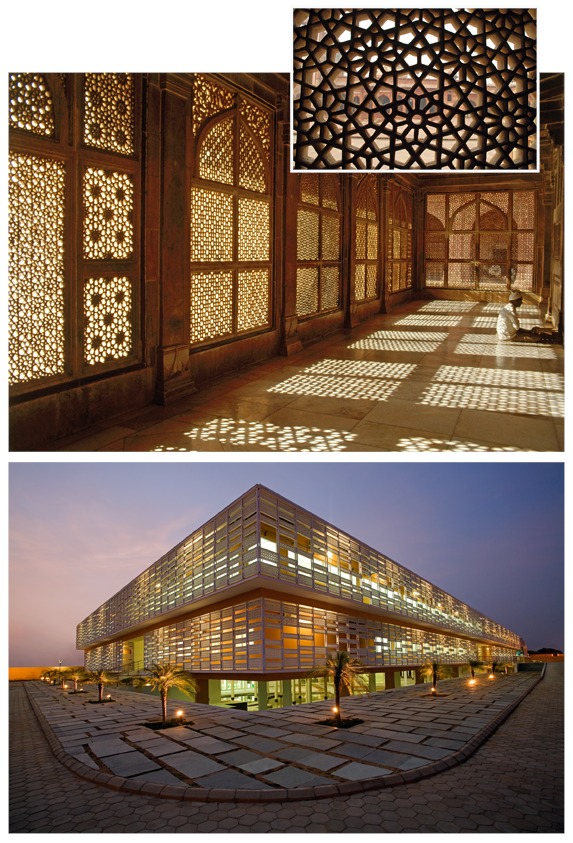
The *jaali* is an ornamental perforated screen that filters sunlight and reduces solar heat gain. This design element, prevalent in Indian and Middle Eastern architecture, is seen here at the Tomb of Sheikh Salim Chishti (top), built in the late 1500s in the city of Fatehpur Sikri, India, and at Pearl Academy (bottom). Clockwise from bottom: © André J. Fanthome; © ephotocorp/Alamy; © Dinodia Photos/Alamy

India and other nations in South Asia and Southeast Asia are on track to record the world’s biggest increases in demand for air conditioning. Sales of air conditioners in India rose an estimated 17% over the past three years, with sales rising fastest among residential users.^^6^^ Michael Sivak, a research professor at the University of Michigan, estimated the potential demand for cooling in Mumbai alone at about 24% of the entire U.S. demand.^^4^^

Mohammad Arif Kamal, an assistant architecture professor at Aligarh Muslim University, explains that air conditioning has become *de rigueur* in India. “Air conditioning has become a social and status symbol,” he says. “People are discarding their old, traditional homes made of bricks, mud, adobe, timber, bamboo, etc., in exchange for boxes made of concrete and glass in pursuit of modernization, which consumes a lot of operational energy.”

China, on the other hand, is likely to surpass the United States as the biggest user of electricity for air conditioning by 2020, according to Cox. Michael A. McNeil, a researcher at the Lawrence Berkeley National Laboratory, has looked at global air-conditioning use and found that air-conditioner ownership in warm-climate countries grows more rapidly than does economic growth. And if China is any example, he contended in a 2008 article, the adoption of air conditioning in developing countries could be rapid and vast: In 1990, fewer than 1% of Chinese households owned air conditioners, but by 2003, he reported, that number had increased to 62%.^^7^^

More recently, figures from the Chinese National Bureau of Statistics indicate that in 2011 Chinese consumers bought roughly 110 air conditioners per 100 urban households and roughly 18 units per 100 rural households.^^8^^ Stan Cox, senior research scientist at the nonprofit Land Institute and author of the 2010 book *Losing Our Cool*, predicts that China will surpass the United States as the biggest user of electricity for air conditioning by 2020. According to his “very rough” estimates, total world air conditioning use currently consumes about 1 trillion kilowatt hours (kWh) of electricity annually—more than twice the total energy consumption of the continent of Africa for all purposes, he says—and could expand tenfold by 2050. “Of the trillion kWh, approximately half is consumed currently by the United States, but the great bulk of the projected tenfold increase will be in Asia,” Cox predicts.

## Environmental Impacts of Air Conditioners

In addition to placing strains on nations’ power grids, air conditioners pose threats to the environment and environmental health, primarily as contributors to global warming. “The amount of electricity that’s used for air conditioning is a huge part of an energy load for most countries, and it’s going up,” says Durwood Zaelke, president of the nonprofit Institute for Governance and Sustainable Development. “You’re putting out more climate pollutants as you’re burning more coal or gas to run the air conditioners, and you’re also putting out the greenhouse gases that serve as the refrigerants in the equipment.”

According to Cox, approximately 80% of the impact of air conditioning on climate results from the draw on fossil fuel–fired power plants. The remaining 20% comes from the units’ refrigerants, the liquid agents within the coils that are used to cool and dehumidify the air.

Different types of refrigerants have been used in air conditioners over the years. The discovery that chlorofluorocarbons (CFCs) are major contributors to ozone-layer breakdown^^9^^ prompted an international response that led to the creation of the Montreal Protocol on Substances that Deplete the Ozone Layer, which went into effect in 1989 and eventually eliminated production of CFCs in 1996.^^10^^ The CFCs were replaced by hydrochlorofluorocarbons (HCFCs), a transitional fluorocarbon with a reduced impact on ozone depletion to be used only while companies developed better coolants. Today these replacements are, themselves, being phased out^^11^^ and replaced by hydrofluorocarbons (HFCs).

HFCs have no impact on ozone depletion because they lack chlorine. However, they have been found to possess a characteristic that is not covered by the Montreal Protocol: They are super-greenhouse gases with high potential to contribute heavily to global warming.^^12^^ The effort to solve one environmental problem, therefore, is likely exacerbating another.

Stephen O. Andersen, cochairman of the Technology and Economic Assessment Panel (TEAP) of the Montreal Protocol, says the challenge now is coming up with new refrigerants. One of the more promising candidates, he says, is refrigerant-grade propane, which offers the benefits of energy efficiency and minimal climate impact. But propane-powered air conditioners would need to be installed by trained personnel, unlike traditional window units, and with sales of conventional air-conditioning units continuing at a brisk pace and uneven regulation of HFCs worldwide, there’s been little motivation to try moving them to the marketplace.

Zaelke says a movement has begun among the Montreal Protocol signatories to address HFCs even though they are not ozone depleters, on the grounds that increased HFC emissions are a result of decisions made under the Protocol. The Federated States of Micronesia, concerned about rising sea levels, last year issued a call seeking a phase-down on the production and use of these chemicals.^^13^^ The United States, Canada, and Mexico similarly have called for a formal amendment to the treaty to phase down HFCs.^^14^^ More than 110 of the Protocol’s 197 signatories have shown their support. Clearly, continued movement in that direction would provide a greater stimulus to the search for new refrigerants, as will a measure proposed by the European Commission in November 2012 to reduce HCF emissions in European countries.^^15^^ But when the Montreal Protocol parties met in Geneva in November 2012, a small number of nations blocked progress on the amendments.^^16^^

Meanwhile, engineers are building more efficient air conditioners that draw less heavily on power supplies. For many years the Japanese government has pursued a number of measures to reduce electrical demand, including an initiative called the Top Runner Program.^^17^^ The program sets efficiency standards for electrical products, including air conditioners, by identifying the most efficient ones being produced and establishing that performance level as the new baseline that other manufacturers must match.

Although energy efficiency provides many benefits that make it worthwhile to pursue. Zaelke and Cox note, that improving the operating efficiency of air conditioners may have less impact on reducing global warming than one might anticipate. “There’s this rebound effect,” Zaelke says. “You improve the efficiency by fifty percent, so people say, ‘It only costs me half as much, so I can use more.’”

Cox provides numbers to support this assessment: He says residential air-conditioning units used in the United States between 1993 and 2005 increased in overall efficiency by 28%—which he calls “pretty significant”—but the amount of energy used to cool the average air-conditioned U.S. home went up 37% during those same 12 years.^^18^^

## Calling on Traditional Technologies

Although air-conditioning use will certainly continue to increase globally with no serious regulatory frameworks in sight, some observers believe awareness of its environmental impact is beginning to change the ways in which architects and engineers, at least, are approaching the challenge of keeping people cool. In fact, many planned and existing buildings employ a variety of technologies—new and old—to achieve comfortable indoor temperatures without resorting to the use of air conditioners.

Pablo LaRoche, a professor of architecture at California State Polytechnic University Pomona who also practices in the Los Angeles firm HMC Architects, believes the true solution for temperature management is passive cooling systems. Such a system transfers heat from a building to any combination of exterior heat sinks—such as the air, water, and earth—through special design details in the building itself. By providing pathways to carry heat from the interior of the building to the outdoors, he explains, the building itself becomes the air conditioner, using little or no energy at all.^^19^^

LaRoche points out that different types of passive cooling systems work better in different climates. For example, he says evaporative cooling^^20^^ (which adds moisture to the air) works best when the air is dryer, whereas night flushing^^21^^ (using cold night air to ventilate a building and cool its thermal mass) is preferable for places where there is a greater temperature difference between daytime and nighttime temperatures.

Passive downdraft evaporative cooling (PDEC) employs the spraying of microscopic water droplets into the air, a concept borrowed from traditional architecture in Pakistan, Iran, Turkey, and Egypt, according to Kamal. These traditional buildings were topped by wind-catching hoods (*malqafs*) that pulled air down chimneys and cooled it by directing it across a source of moisture: a pool, a fountain, or porous pots that seeped water. Contemporary PDEC buildings also employ wind catchers but replace the water pots with wet cellulose pads or similar devices. Kamal cites the Torrent Research Centre in Ahmedabad, India, as an excellent example of contemporary use of PDEC. The center was completed in 1999 and has reportedly provided comfortable conditions for occupants while also recording extremely low energy consumption.^^22^^

Another example of a hot-climate structure using water and traditional design for cooling is Pearl Academy in Jaipur, India. The building includes a sunken courtyard pool, which architect Manit Rastogi explains functions similarly to a basement, staying cooler than the aboveground air in the summer and warmer in the winter; breezes flowing under the raised building create evaporative cooling currents that push air up through atria and open stairwells.

The building also features an exterior latticed screen ( *jaali* ) enveloping the building, a traditional feature of Rajasthani architecture that provides a thermal buffer for buildings (however, this is not considered true passive cooling, but rather a strategy to avoid overheating). Despite the fact that the building is located in a hot desert climate, Rastogi says it maintains interior temperatures of 80–85°F even when it’s 110°F outside, using minimal mechanical air conditioning just two months of the year.^^23^^

One of the most unusual and innovative examples of a structure utilizing traditional technology might be architect Mick Pearce’s Eastgate Centre, a shopping center in Harare, Zimbabwe, that was inspired by a 1992 BBC television program on termites, hosted by naturalist David Attenborough.^^24^^ Pearce was struck by the termites’ use of the thermal capacity of the ground and the mound, and their labyrinths of ventilation tunnels. “The termite mound which we see above ground is a breathing and air-conditioning system like the human lung,” he says.

Eastgate Centre relies on night flushing: Cool night air is driven through a multitude of air passages within the building’s heavy concrete and masonry structure, cooling the concrete vaulted ceiling, which absorbs heat during the day. The accumulated heat from each day is vented at night through these same passages, partly by fans and partly by convection forces in 48 huge stacks that run through the center of the building.

Pearce says it took about three years to optimize the timing of the daytime and nighttime fans to align with diurnal differences in temperature. “It was like tuning an organ built into a church, where the building resonance is important,” he says. “Another factor was the occupation of the building, where—like the termitary—the occupants’ heat [output] is crucial to the cycles.” According to Pearce, Eastgate uses 10% of the energy of comparably sized air-conditioned buildings in Harare.

Still another scheme for alternative cooling has been in place in Toronto for eight years: a “deep water source cooling” system in which cool water is pumped from a five-kilometer depth in Lake Ontario to participating office buildings and through metal coils.^^25^^ Fans blow the cool air from the coils into the buildings’ climate-control systems, reducing their energy demands. Although mostly used in cooler climates, it is also being explored in warmer areas. A project using this technology is about to break ground in Honolulu and will use seawater.^^26^^

## Small-Scale Fixes

Apart from these large-scale demonstrations that mechanical air conditioning can be eliminated or reduced, experts say there are many smaller ways that workplaces and homes can be made comfortable during hot weather without air conditioning.

Leon Glicksman, a professor of building technology and mechanical engineering at the Massachusetts Institute of Technology, points to ceiling fans as an additional cooling mechanism that’s much cheaper than air conditioning. The solar loads on both office buildings and homes can be lowered by using exterior shades and awnings to keep the building from overheating. And he says roofs can be painted white to deflect heat.^^27^^ Glicksman is also developing design programs for natural ventilation where cross ventilation and vertical ducts used as chimneys can be used in the spring and fall to enhance airflow through the buildings and reduce the use of air conditioning. Other time-tested ways by which people can stay cool while saving money and reducing energy use include installing window awnings and exterior roll blinds to block sunlight from hitting the house, reducing heat gain from attics through the use of thorough insulation and installation of roof vents, closing windows and doors during the day to block out the heat and opening them at night, installing ceiling fans, and reducing the use of heat-generating appliances.^^28^^

The problem with employing alternative cooling techniques and strategies in the workplace, however, is that many existing buildings aren’t designed to accommodate them. The Japanese government’s efforts to reduce electrical demand included a recommendation that office buildings set air conditioners no lower than 82°F. When energy conservation became even more pressing after the Fukushima Daiichi disaster of March 2011, the government instituted a “Super Cool Biz” campaign to encourage ending dress codes calling for jackets and ties and wearing light, cool clothing instead.^^29^^

While 82°F might sound pretty hot for an office setting, Glicksman says there’s been research that shows an adaptive effect that takes into account the nature of the work surroundings. That is, in a building where air is circulating or the workers can open windows and have some control over their environment, the upper tolerable temperature is higher than in “a sealed-up box where they’re at the mercy of whatever the air-conditioning system is doing.”^^30^^

**Figure:**
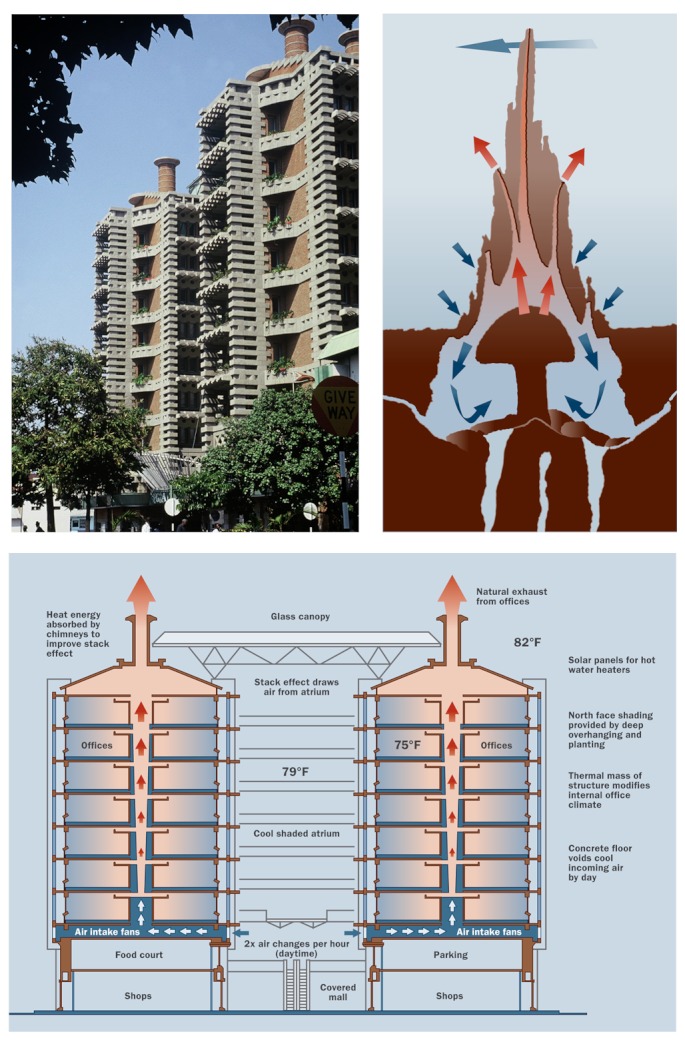
The ventilation passages and chimneys of a termite hill (near left) were the inspiration for Harare’s Eastgate Centre, where fans and convection are used to exhaust heat buildup from the complex (below). Cool nighttime air chills the masonry mass of the building, which absorbs heat during the daytime. The jagged concrete facing, interspersed with greenery, is designed to absorb the least amount of heat during the day and release the most during the night (far left). Photograph: © David Brazier. Illustrations by Daniel Gallant/Foundry Zero. Adapted from artwork courtesy of Mick Pearce.

In fact, cultural acceptance of air conditioning varies widely. They’re very rare in French homes and not that common in Spanish ones either, says Lloyd Alter, an adjunct professor at Canada’s Ryerson University School of Interior Design. “In France, they think air conditioners make you sick,” he explains. “In Spain, their culture revolves around being outside and taking advantage of it: ‘We go out and eat our dinner at 10 o’clock at night, and we take it easy mid-day.’”

## Looking to the Future

Zaelke sees a future in which governments play a stronger role in setting manufacturing standards, as the Japanese are with their Top Runner program; tax credits to stimulate innovative technology; and comprehensive labeling programs, somewhat like the LEED (Leadership in Energy and Environmental Design) ratings system developed by the U.S. Green Building Council, to address elements of air conditioning beyond the energy efficiency covered by federal Energy Star ratings. He also thinks part of the answer is returning to a design mindset that was prevalent before the advent of air conditioning. “Before cheap energy, we used to do a better job designing our buildings,” he says. “For example, we used to know how to situate a building so you had deciduous trees providing shade during the summer and evergreens providing shelter from the wind.”

Pearce asserts that air conditioning has made architects lazy. “Air conditioning has allowed them to design buildings based on formal concepts without any response to the natural environment,” he says. “Architects should design buildings whose form is shaped by a scientific understanding of natural processes at the building’s location and not by some purely whimsical sculptural shape.”

**Figure:**
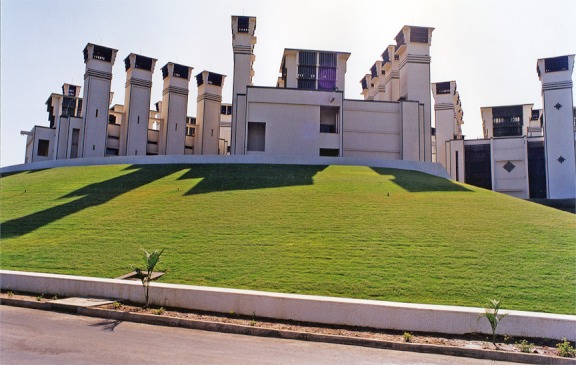
The Torrent Research Centre of Ahmedabad, India, uses wind-catching intake towers to pull in air and cool it by diverting it through a fine mist. The cooled air descends through an open central corridor and is drawn into work spaces on each level. Exhaust towers around the perimeter of the complex vent hot air at night. Abhikram

LaRoche believes it’s imperative for people in his profession to pursue minimal environmental impacts when designing structures and strive to incorporate alternative ways to cool them. He says HEED (Home Energy Efficient Design)—free software developed at the University of California, Los Angeles—is a good example of a residential energy design tool that can be used by anybody.^^31^^ “Tools such as this one help any homeowner or designer produce low-energy buildings,” he says.

Ultimately, LaRoche says, architectural education is key to change: “If the new architects aren’t trained in the design of low-carbon, low-energy buildings, nothing will happen. New students must be trained with new software and tools that we did not have just a couple of years ago.” He adds, “Whenever we do passive cooling in a building instead of mechanical cooling, we’re helping our planet. It’s also good for our pockets, and our buildings are more culturally responsive to the environment around them.”

## Nothing New under the Sun

Biomimicry research looks to nature for inspiration and guidance in developing new technologies. The most recent Student Design Challenge at the nonprofit Biomimicry 3.8 Institute, in Missoula, Montana, had climate change as a theme and produced a number of innovative cooling designs inspired by nature. The first-place winners, a group of students from Art University of Isfahan, Iran, designed a hot-weather structure inspired by desert snails and their naturally cooling shells. Second place went to a group of students from the University of Latvia who devised a shading system that mimics how flowers and stomata open and close in response to sunlight.

**Figure:**
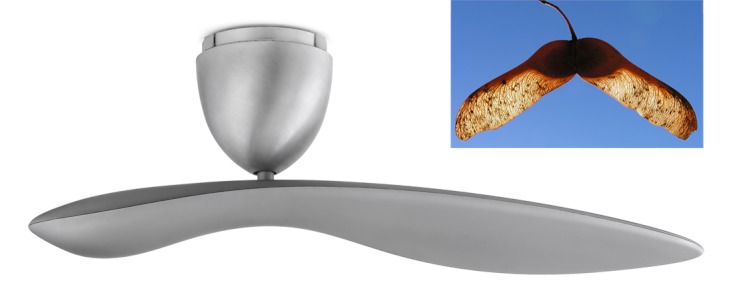
“Whirlybird” seed-inspired blades reportedly enable the Sycamore Ceiling Fan to deliver greater airflow at lower operating speeds than traditional ceiling fans. © Anette Linnea Rasmussen/Shutterstock

Other nature-inspired cooling designs include:

- The Sycamore Ceiling Fan. Designed by two Australian industrial designers, the Sycamore ceiling fan is a single-blade fan inspired by the flight of falling sycamore tree pods. The design—one end of the blade is much larger than the other—provides a more efficient means of moving the air, the designers say.
- The COMOLEVI Forest Canopy. Lofsee Company, Ltd., designed an artificial shade canopy to mimic natural tree branches and leaves. Its advantage: It provides cooling along with pleasant, dappled sunlight.
- The Homeostatic Façade System. The New York design firm Decker Yeadon used muscles and homeostasis in biological systems as the inspiration for their double-skin glass façade system for large buildings. The façade resembles a window with a swirling design that opens and closes itself in response to changes in interior room temperature to permit or deny heat gain from the sun.

**Figure:**
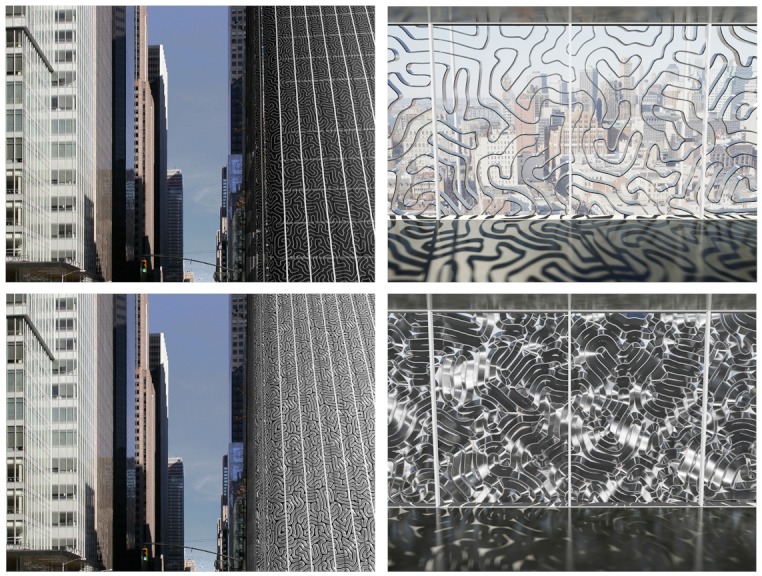
The prototype Homeostatic Façade System consists of squiggles of flexible core wrapped in a dielectric elastomer. The elastomer expands and contracts in response to environmental cues such as temperature, changing the shape of the core as it does so and allowing more or less light to enter. Sycamore Technology; Decker Yeadon LLC
